# Tensile Properties and Fracture Behavior of Aluminum Alloy Foam Fabricated from Die Castings without Using Blowing Agent by Friction Stir Processing Route

**DOI:** 10.3390/ma7032382

**Published:** 2014-03-21

**Authors:** Yoshihiko Hangai, Hiroto Kamada, Takao Utsunomiya, Soichiro Kitahara, Osamu Kuwazuru, Nobuhiro Yoshikawa

**Affiliations:** 1 Graduate School of Engineering, Gunma University, Kiryu 376-8515, Japan; E-Mail: t08302021@gunma-u.ac.jp; 2 SIT Research Laboratories, Shibaura Institute of Technology, Saitama 337-8570, Japan; E-Mail: utunomiy@sic.shibaura-it.ac.jp; 3 Hokudai Co., Ltd., Abira 059-1434, Japan; E-Mail: soichiro_kitahara@hokudai-jp.com; 4 Graduate School of Engineering, University of Fukui, Fukui 910-8507, Japan; E-Mail: kuwa@u-fukui.ac.jp; 5 Institute of Industrial Science, The University of Tokyo, Tokyo 153-8505, Japan; E-Mail: yoshi@telu.iis.u-tokyo.ac.jp

**Keywords:** cellular materials, foam, friction stir welding, die casting, X-ray computed tomography (CT)

## Abstract

Al foam has been used in a wide range of applications owing to its light weight, high energy absorption and high sound insulation. One of the promising processes for fabricating Al foam involves the use of a foamable precursor. In this study, ADC12 Al foams with porosities of 67%–78% were fabricated from Al alloy die castings without using a blowing agent by the friction stir processing route. The pore structure and tensile properties of the ADC12 foams were investigated and compared with those of commercially available ALPORAS. From X-ray computed tomography (X-ray CT) observations of the pore structure of ADC12 foams, it was found that they have smaller pores with a narrower distribution than those in ALPORAS. Tensile tests on the ADC12 foams indicated that as their porosity increased, the tensile strength and tensile strain decreased, with strong relation between the porosity, tensile strength, and tensile strain. ADC12 foams exhibited brittle fracture, whereas ALPORAS exhibited ductile fracture, which is due to the nature of the Al alloy used as the base material of the foams. By image-based finite element (FE) analysis using X-ray CT images corresponding to the tensile tests on ADC12 foams, it was shown that the fracture path of ADC12 foams observed in tensile tests and the regions of high stress obtained from FE analysis correspond to each other. Therefore, it is considered that the fracture behavior of ADC12 foams in relation to their pore structure distribution can be investigated by image-based FE analysis.

## 1. Introduction

Al foam is a lightweight material with high energy absorption, high sound insulation, and high thermal conductivity [1,2]. Therefore, Al foam has been used in a wide range of applications, such as automotive components, railway components, and building materials [2,3]. One of the promising processes for fabricating Al foam involves the use of a foamable precursor [2–9]. In this process, an Al alloy composite that contains a uniformly distributed blowing agent powder, called a precursor, is fabricated. Upon heat treatment of the precursor, gases generated by the decomposition of the blowing agent expand the softened Al alloy to fabricate Al foam. Several routes have been proposed for fabricating the precursor, such as the powder metallurgical (P/M) route [3–5], accumulative roll-bonding (ARB) route [6], compressive torsion processing route [7], and friction stir processing (FSP) route [8].

As an alternative to using a blowing agent, which is relatively expensive and an explosion hazard, some researchers have fabricated the precursor by the P/M route without the use of a blowing agent [10–13]. The expansion of Al alloy occurred upon heating a precursor that had previously contained a large amount of gases during the P/M process [10,11], or upon heating a precursor produced by the compaction of Al powder without the addition of any further materials, while changing the ambient pressure [12,13]. Recently, a simpler and more cost-effective process using the FSP route without a blowing agent has been developed [14,15]. In this processing route, Al alloy high-pressure die castings, which have a high gas porosity [16,17] are used as the starting material. This gas porosity can be used to induce foaming as an alternative to a blowing agent. Moreover, the FSP route has the advantages of reducing the cost of Al foam and lower environmental impact. In addition, FSP is a simple and short-duration process with potentially high productivity [18,19]. In addition, it does not require time-consuming heating processes that consume a large amount of energy [18,19] to fabricate the precursor. FSP can also induce intense stirring, by which powder can be easily mixed into an Al plate [18–21] and segregated microstructures and gas pores can be easily and uniformly mixed [22,23], by simply traversing the rotating tool used in FSP. Furthermore, inexpensive and high-recyclability Al alloy die-casting plates can be used as the starting material instead of expensive Al alloy powder. It has been shown that a porosity of approximately 50%–80% can be achieved using Al alloy die castings, which contain a large number of gas pores, without the use of a blowing agent [15]. In addition, the pores of the Al foam obtained using die castings without a blowing agent are smaller and have higher sphericity than those of Al foam fabricated using a blowing agent [15,24–26].

Traditionally, Al foam has been used as a sandwich structure, which is a composite consisting of an Al foam core with two dense metallic face sheets [2,3,27–29], because Al foam has inferior tensile and bending strength. It is expected that the Al foam fabricated from Al alloy die castings without a blowing agent can be used to fabricate thin Al foam plates [30] owing to the small pores and good mechanical properties of die-casting Al alloys. The non usage of face sheets can decrease the weight of the components and the number of fabrication processes, resulting in increased cost-effectiveness and increase recyclability. Good understanding of the tensile and bending properties of the Al foam, fabricated from Al alloy die castings without a blowing agent, is necessary for its successful industrial application.

In this study, the pore structure and tensile properties of Al foams fabricated from Al alloy die castings without a blowing agent by the FSP route were investigated. First, X-ray computed tomography (X-ray CT) observation was carried out to characterize the pore structure of the tensile test specimens before conducting tensile tests to confirm that the Al foams fabricated in this study also have small pores. Next, the tensile properties of the fabricated Al foams were investigated and the effect of the porosity on the tensile properties was also examined. The results were compared with the tensile properties of commercially available ALPORAS (Shinko Wire Co., Ltd., Tokyo, Japan) [31]. In addition, image-based finite element (FE) analysis of the tensile tests on the fabricated Al foam was conducted. A detailed three-dimensional FE model was reconstructed from the X-ray CT images of the obtained Al foam. In the image-based FE analysis, the whole of a specimen subjected to a tensile test can be considered using an analytical model with the actual three-dimensional complicated pore structure of Al foam. The result of the FE analysis was compared with the actual tensile test conducted in this study to investigate the effect of the pore structure distribution in the specimen on the fracture behavior of the Al foam.

## 2. Results and Discussion

### 2.1. Tensile Tests on ADC12 foams

**Figure 1.**
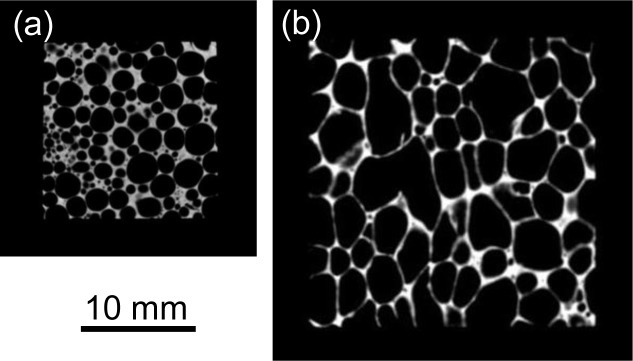
Two-dimensional cross-sectional X-ray CT images of tensile test specimens: (**a**) ADC12 foam (*p* = 70.3%); and (**b**) ALPORAS (*p* = 88.0%).

Figure 1a,b show typical two-dimensional cross-sectional X-ray CT images of tensile test specimens of ADC12 foam (*p* = 70.3%) and ALPORAS (*p* = 88.0%). In the X-ray CT images, gray regions indicate the cell walls of ADC12 foam and black regions indicate pores and background air. It can be seen that the ADC12 foam had smaller pores with higher sphericity than those of ALPORAS.

**Figure 2.**
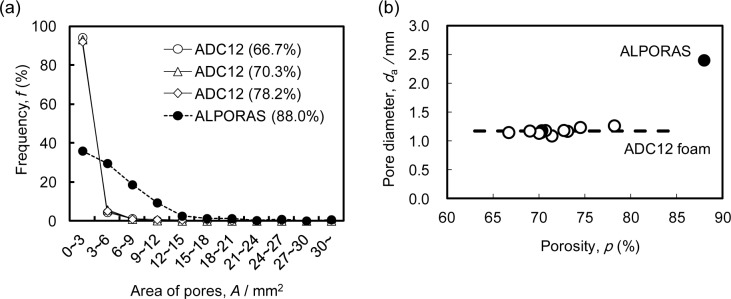
(**a**) Relative frequency of pore area *A* for ADC12 foams and ALPORAS; (**b**) Relationship between porosity *p* of the foams and average pore diameter *d*_a_.

Figure 2a shows the relative frequency of the pore area *A*, which was evaluated from two-dimensional cross-sectional X-ray CT images such as that shown in Figure 1, for typical three ADC12 foams with *p* = 66.7% (*i.e*., the minimum porosity obtained in this study), 70.3% and 78.2% (*i.e*., the maximum porosity obtained in this study), along with ALPORAS with *p* = 88.0%. For the ADC12 foams, more than 80% of the pores had an area of less than 3 mm^2^, and the frequency of pores rapidly decreased with increasing pore area. This tendency was observed for all the ADC12 foams regardless of the porosity. In contrast, for ALPORAS, approximately 40% of the pores had an area of less than 3 mm^2^, and the frequency of pores gradually decreased with increasing pore area. Therefore, the pores in the ADC12 foams were smaller and had a narrower distribution than those in ALPORAS.

Figure 2b shows the relationship between the porosity of the foams and the average pore diameter *d*_a_ for eleven ADC12 foams along with ALPORAS. The variation of the porosity was within ±7% for the samples fabricated from ADC12 plates under the same die-casting conditions. This variation is considered to be due to the variations in the amount of gases contained in the plates during the die-casting process. It can be seen that the average pore diameters for the ADC12 foams were almost the same regardless of the porosity and were smaller than that for ALPORAS. These results are consistent with a previous study [15].

**Figure 3.**
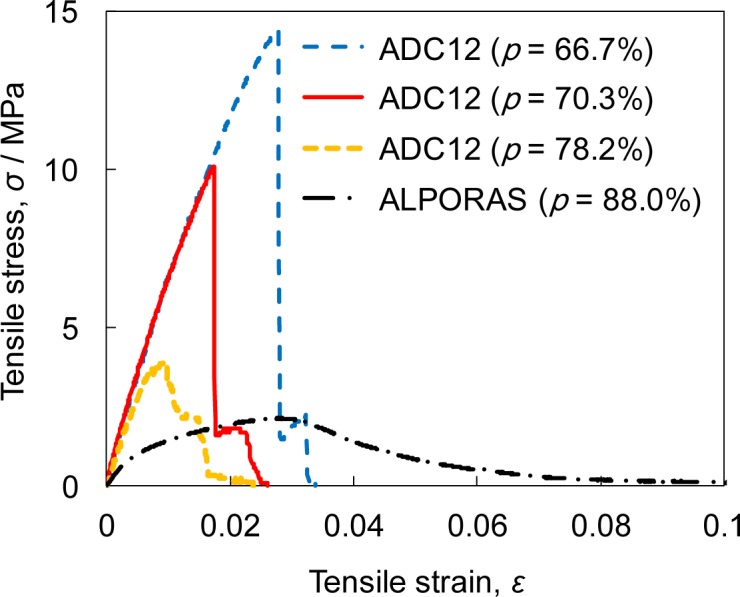
Relationship between tensile strain and tensile stress of ADC12 foams and ALPORAS.

Figure 3 shows the stress-strain curves obtained by tensile tests on ADC12 foams with *p* = 66.7%, 70.3%, and 78.2%, along with ALPORAS with *p* = 88.0%. For the ADC12 foams, as the strain increased, the stress rapidly increased and fracture occurred immediately after the tensile stress reached its maximum value. Namely, the ADC12 foams exhibited brittle fracture, which is attributed to the Al-Si eutectic nature of the ADC12 Al alloy [32–34] used as the base material and the presence of oxide films. This brittle fracture of ADC12 foams is consistent with the compression tests reported in previous studies [15,24–26]. The Young’s modulus of the ADC12 foams slightly decreased as the porosity increased but remained almost constant for the range of porosities of the ADC12 foams obtained in this study. In addition, as the porosity of the ADC12 foams increased, the tensile strength decreased. In contrast, for ALPORAS, as the strain increased, the stress gradually increased and local fracture of the cell wall began to occur immediately after the tensile stress reached its maximum value. The Young’s modulus of ALPORAS was lower than that of the ADC12 foams. After crack initiation, the tensile stress gradually decreased with increasing tensile strain owing to crack propagation by the continuous fracture of the cell wall, then the final fracture occurred. Namely, ALPORAS exhibited ductile behavior owing to the ductile nature of the pure Al base material.

**Figure 4.**
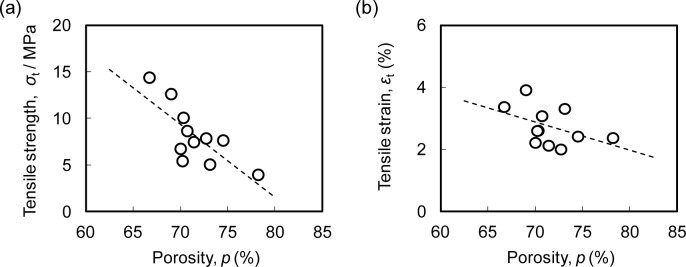
Tensile properties of ADC12 foams. (**a**) Relationship between porosity of the ADC12 foams and tensile strength σ_t_; (**b**) Relationship between porosity of the ADC12 foams and tensile strain ε_t_.

Figure 4a,b show the relationships between the porosity *p* and the tensile strength σ_t_ and tensile strain ε_t_, respectively, for the ADC12 foams. Although there was some variation, it was shown that the tensile strength and tensile strain tend to decrease with increasing porosity. The variation in the tensile strength and tensile strain was caused by the variation of the pore structure distribution in the specimen due to the segregation of gases in the die-casting plates used as the starting material as well as by inhomogeneous foaming. The variation of the pore structure distribution may be reduced by optimizing the die-casting conditions used to fabricate die-casting plates and the FSP conditions used to homogeneously mix the gas pores in the die-casting plates. Another cause of the variation in the tensile strength and tensile strain was the very large number of micropores in the cell walls of the Al foams, which have a diameter ranging from several μm to several tens of μm, and a mean diameter of 3.6 μm [35]. These micropores cannot be observed by the X-ray CT used in this study owing to the resolution of the obtained images [36]. The distribution of these micropores is considered to affect the fracture of the cell walls. Moreover, cell walls made from ADC12 die-casting Al alloy contain many inclusions and oxide films, which become the initiation of fracture [32–34], which is commonly observed in ADC12 die-casting products. The segregation of these inclusions may affect the fracture of the cell walls.

### 2.2. Image-Based FE Analysis of Tensile Tests on ADC12 Foams

**Figure 5.**
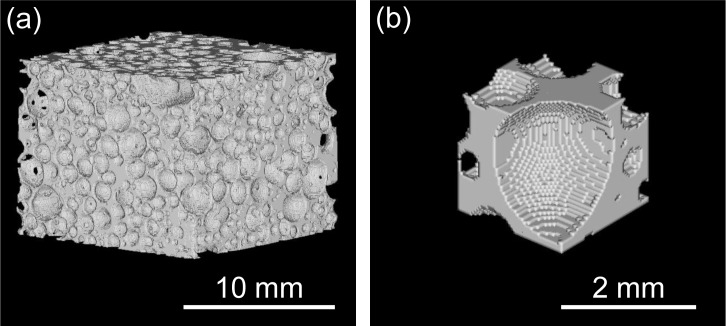
(**a**) Reconstructed three-dimensional voxel model of ADC12 foam tensile test specimen (*p* = 70.3%); (**b**) Enlarged voxel model of ADC12 foam tensile test specimen around some of the pores in Figure 5a.

Figure 5a shows the reconstructed three-dimensional voxel model of an ADC12 foam tensile test specimen with *p* = 70.3%, in which only the aluminum voxels were visualized. It was shown that an FE analysis model of the complicated pore distribution of ADC12 foam can be fabricated. Figure 5b shows a three-dimensional voxel model of the area around some of the pores, which was extracted and enlarged from Figure 5a. It is apparent that each pore is separated by sufficiently fine voxels.

**Figure 6.**
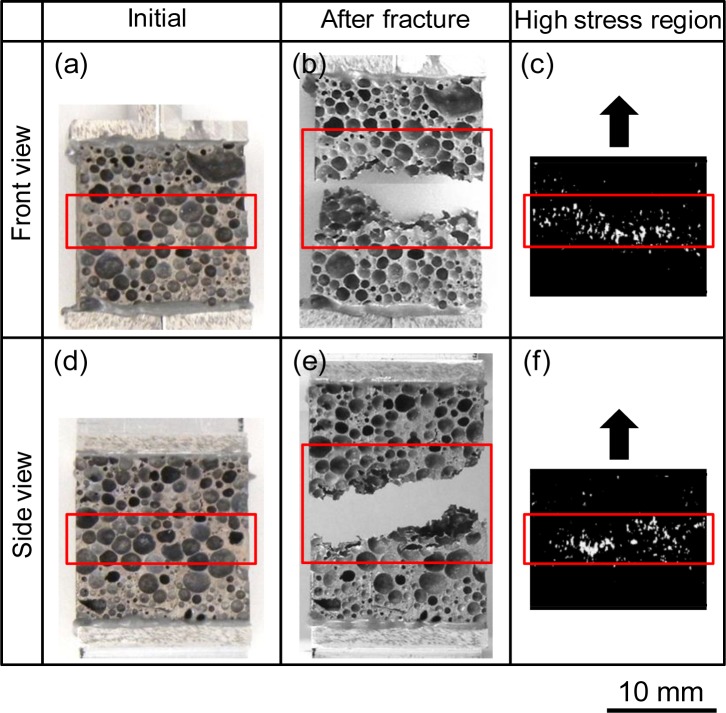
Tensile test behavior and corresponding FE analysis of ADC12 foam (*p* = 70.3%) showing front and side views: (**a**) and (**d**) initial pore structure; (**b**) and (**e**) pore structure after fracture; and (**c**); and (**f**) Mises equivalent stress distributions obtained by image-based FE analysis.

Figure 6 shows the tensile test result and the corresponding FE analysis for the ADC12 foam with *p* = 70.3%, which were observed from the front view and right view. Figure 6a,b show the initial tensile test specimen before the test and Figure 6b,e show the tensile test specimen after the test, which exhibited fracture. The red boxes in the figures indicate the regions exhibiting fracture. As can be seen, fracture occurred almost perpendicular to the tensile load direction but with a slight incline. Figure 6c,f respectively show the high stress regions for the specimen in Figure 6a,d obtained by image-based FE analysis of the tensile test. The white regions indicate voxels with Von Mises equivalent stress greater than five times the mean Von Mises equivalent stress of all the voxels in the ADC12 foam. Voxels with comparatively high stress are mainly distributed in the red boxes, which are the same regions as those indicated in Figure 6a,d. It was shown that there is a clear correspondence between the fracture path and the regions exhibiting high stress. This trend was also apparent in most other test specimens. The above observations indicate that by using image-based FE analysis to observe the stress distribution, it is possible to investigate the effect of the pore structure distribution in the specimen, at least qualitatively, on the fracture behavior observed in tensile tests on ADC12 foams.

Although it is possible that fracture occurred as a result of the segregation of inclusions and micropores in the cell walls, it is assumed that the fracture of the ADC12 foams in the tensile tests mainly occurred by the concentration of stress induced by the pore structure, as can be seen from the image-based FE analysis. Clearly, further studies are necessary to reveal the effect of the pore distribution and the microstructure of the cell walls on the mechanical properties of ADC12 foams.

## 3. Experimental Section

### 3.1. FSP Procedure

**Figure 7.**
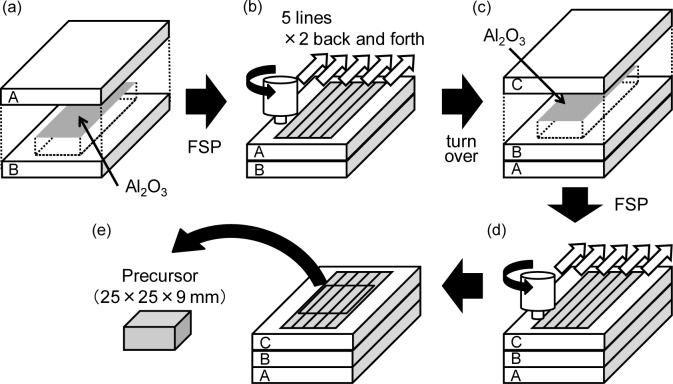
Schematic illustration of the fabrication process of the precursor of ADC12 foam from ADC12 Al alloy die-casting plates by FSP route.

Figure 7 shows a schematic illustration of the fabrication process of the precursor by the FSP route. As the starting material for the fabrication of the Al foams, as-received Al-Si-Cu Al alloy ADC12 (equivalent to A383.0 Al alloy) die-casting plates of 3 mm thickness, 80 mm width and 210 mm length were used. Table 1 shows the amounts and types of gases in the die-casting plates, which were measured by gas chromatographic analysis after melting the ADC12 die-casting plates. The source of N_2_ gas is considered to be gases derived from air existing in the cavity, the runners and the injection system. In contrast, the source of H_2_ and other gases such as CH_4_ is considered to be the reaction gases formed when the melted Al alloy encounters the parting and lubricant agent.

As shown in Figure 7a, two plates were stacked with alumina powder (α-Al_2_O_3_, ~1 μm) distributed between them as the stabilization agent. The mass of alumina used was 5% of that of the Al alloy with dimensions of the area over which the alumina was distributed and the length of the tool probe. As shown in Figure 7b, FSP was carried out using a 1D-FSW machine (Hitachi Setsubi Engineering Co., Ltd., Ibaraki, Japan). The FSP tool has a cylindrical shape with a screw probe. The diameter of the tool shoulder was 17 mm, the diameter of the tool probe was 6 mm and its length was 5 mm. The tool rotation speed was 1000 rpm, and the speed of the tool as it traversed the plate was 100 mm/min throughout the experiments. A tilt angle of 3 deg was used. The multipass FSP technique [37–39] (5 lines × 2 times back and forth, *i.e*., the FSP region was stirred four times) was applied to obtain a larger amount of precursor and to mix the segregated gases and alumina powder thoroughly. As shown in Figure 7c, the plate was turned over, and alumina was placed on the reverse side of the FSP surface along the path of the FSP tool. Finally, as shown in Figure 7d, the same FSP procedures as those shown in Figure 7b were carried out once again to obtain a thicker precursor.

### 3.2. Foaming Procedure

Precursor samples of 25 mm width, 25 mm length and 9 mm thickness were machined from the region subjected to FSP as shown in Figure 7e. The precursor sample was then heated in a preheated electric furnace. The holding temperature (equal to the preheating temperature) and the holding time during the heating process were fixed at 948 K and 12–14 min, respectively, with reference to a previous study [15]. Then, the obtained ADC12 foams were cut by electro-discharge machining to fabricate cubic tensile specimens. Each specimen of ADC12 foam had a side of 15 mm. Eleven tensile test specimens with porosities ranging from 66.7% to 78.2% were obtained. In addition, an ALPORAS cubic tensile test specimen with a side of 25 mm was fabricated with a porosity of 88.0% from an as-received ALPORAS block. The size of each specimen was selected so that there were at least seven pores on each face of the specimen to suppress the edge effect.

### 3.3. Tensile Test Procedure

The grip part used for the tensile tests was realized by bonding a jig using an EW2010 structural adhesive (Sumitomo 3M Limited, Tokyo, Japan) to the surface of the ADC12 foam. Tensile tests were carried out at room temperature in ambient air using an Autograph AG-100kNG universal testing machine (Shimadzu Corporation, Kyoto, Japan). The tensile strain rate was set as 1.67 × 10^−3^ s^−1^.

### 3.4. X-Ray CT Observation Procedure

Before the tensile tests were conducted, the pore structure in the ADC12 foams and ALPORAS tensile test specimens with the bonded jig were observed nondestructively by X-ray CT using an SMX-225CT microfocus X-ray CT system (Shimadzu Corporation, Kyoto, Japan) at room temperature.

The X-ray tube voltage and current were 80 kV and 30 μA, respectively. The areas *A* and average diameters *d*_a_ of the pores were evaluated from two-dimensional cross-sectional X-ray CT images using WinROOF image processing software (Mitani Corporation, Tokyo, Japan). An appropriate threshold was set to distinguish the Al and the pores, in which the cell walls of pores were thickened to prevent the loss of walls thinner than one voxel. Pore volumes were slightly underestimated as a result, and binarized X-ray CT images were established for evaluation. Pores with areas of less than 0.4 mm^2^ were excluded owing to the resolution of the X-ray CT images [36].

The porosity *p* (%) of the tensile test specimens was calculated as:

$$p(\%)=(\rho_{\text{precursor}}-\rho_{\text{specimen}})/\rho_{\text{precursor}}\times 100$$

where ρ_precursor_ is the density of the precursor before heating, which was evaluated by Archimedes’ principle, and ρ_specimen_ is the density of the tensile test specimen, which was evaluated from the weight and dimensions of the specimen.

### 3.5. Image-Based FE Analysis

An FE analysis model was created by voxel modeling based on the acquired X-ray CT images with reference to previous studies [40]. VOXELCON 2011 image-based structural analysis software (Quint Corporation, Tokyo, Japan) was used for three-dimensional image processing and FE analysis. First, cross-sectional two-dimensional X-ray CT images of the entire specimen, except for several X-ray CT images in the vicinity of the tensile test jigs that included bonding adhesive filled the surface pores of the specimen, were layered to obtain three-dimensional images. Next, an appropriate threshold was set so that the Al alloy and pores were distinguished, and the image voxels were classified into material voxels and space voxels. The noise of X-ray CT images was eliminated using a mean filter. Finally, the material voxels were converted into cubic finite elements, with one voxel corresponding to one element, to obtain a three-dimensional FE analysis model.

A completely fixed bottom surface and 1 mm tension of the upper surface were applied in the FE analysis model as the displacement boundary conditions. A linear elastic FE analysis was performed on the cell walls, assuming that they were ADC12 Al alloy with a Young’s modulus of *E* = 71.1 GPa [41] and a Poisson’s ratio of *ν* = 0.3. The total number of elements in the obtained FE analysis model was approximately 10^7^. The elastic-plastic FE analysis of this large model could not be conducted owing to the limited computational capacity. In addition, in previous studies, the stress distribution obtained from image-based linear elastic FE analysis correctly indicates the layer where compression deformation first occurs according to the results of actual compression tests on ALPORAS (*i.e*., approximately pure Al) specimens [40]. To take into account the fact that ADC12 is a brittle Al alloy [32,33], it is considered that linear elastic FE analysis is sufficient to investigate the fracture behavior observed in tensile tests on ADC12 foam.

To eliminate the effect of the numerical stress concentration (excessive stress) due to surface irregularities in the voxel model, the resultant stress on each voxel of interest was taken to be the arithmetic mean of that of the voxel of interest and those of the 26 three-dimensionally adjacent voxels, *i.e*., a total of 27 voxels. However, at areas such as pore surfaces, which were not surrounded by 26 voxels, the mean value was only calculated using material voxels.

## 4. Conclusions

In this study, ADC12 foams with porosities of 67%–78% were fabricated from Al alloy die castings without using a blowing agent by the FSP route precursor foaming method. The pore structure and tensile properties of the ADC12 foams were investigated and compared with those of commercially available ALPORAS. From the X-ray CT observations of the pore structure of ADC12 foams, it was found that they have smaller pores with a narrower distribution than those in ALPORAS. The tensile tests on ADC12 foams indicated that as their porosity increased, the tensile strength and tensile strain decreased, with strong relations between the porosity, tensile strength and tensile strain. ADC12 foams exhibited brittle fracture, whereas ALPORAS exhibited ductile fracture, which is due to the nature of the Al alloy used as the base material of the foams. By image-based FE analysis using X-ray CT images corresponding to the tensile tests on ADC12 foams, it was shown that the fracture path of ADC12 foams observed in tensile tests and the regions of high stress obtained from FE analysis correspond to each other. Therefore, it is considered that the fracture behavior of ADC12 foams in relation to their pore structure distribution can be investigated by image-based FE analysis.

## Figures and Tables

**Table 1.** Amounts and types of gases in used ADC12 Al alloy die-casting plates.

Type of gas	H_2_	N_2_	CH_4_	CO	CO_2_	C_2_H_4_	C_2_H_6_	Total
**Content (cm^3^/100 g Al)**	73.9	13.1	19.8	53.0	84.8	5.6	–	250.6
